# Baicalein Enhances the Oral Bioavailability and Hepatoprotective Effects of Silybin Through the Inhibition of Efflux Transporters BCRP and MRP2

**DOI:** 10.3389/fphar.2018.01115

**Published:** 2018-10-26

**Authors:** Peng Xu, Hua Zhou, Ya-Zhuo Li, Zhong-Wen Yuan, Chang-Xiao Liu, Liang Liu, Ying Xie

**Affiliations:** ^1^State Key Laboratory for Quality Research of Chinese Medicines, Macau University of Science and Technology, Taipa, Macau; ^2^Department of Nephrology, Guangdong Provincial Hospital of Traditional Chinese Medicine, Guangzhou, China; ^3^State Key Laboratory of Drug Delivery Technology and Pharmacokinetics, Tianjin Institute of Pharmaceutical Research, Tianjin, China

**Keywords:** ABCC2 (MRP2), ABCG2 (BCRP), baicalein, silybin, pharmacokinetics, liver injury

## Abstract

Although hepatoprotective properties of silybin are well documented, the clinical therapeutic efficacy is limited by its low bioavailability due to absorption rates, extensive phase II metabolism, and biliary excretion. As our previous study indicated that metabolic enzymes may have limited effects on the pharmacokinetic (PK) behavior of silymarin, here, we intended to increase the oral bioavailability and bio-efficacy of silybin through the inhibition of active efflux. In Caco-2 and transfected MDCKII cell models, flavone baicalein significantly inhibited the efflux of silybin as a BCRP and MRP2 inhibitor. In addition, baicalein reduced the biliary excretion index (BEI) and biliary clearance of silybin conjugates in the sandwich-cultured rat hepatocyte (SCH) model, indicating the inhibition of baicalein in biliary excretion of conjugated silybin metabolites. PK study demonstrated that baicalein significantly increased the area under the curve (AUC) and C_max_ of silybin and its conjugates, suggesting enhanced absorption *in vivo*. Moreover, coadministration of silybin with baicalein boosted the liver protective, antioxidant, and anti-inflammatory effects of silybin in the carbon tetrachloride (CCl_4_)-induced liver injury model in comparison with silybin given alone. In summary, efflux transporters play a critical role in the low bioavailability of silybin, while inhibition of breast cancer resistance protein (BCRP) and multi-drug resistance protein 2 (MRP2) by baicalein can significantly increase the absorption and bio-efficacy of silybin, which provides a new combination therapeutic approach for the treatment of chronic liver diseases.

## Introduction

Silymarin, a standardized extract of flowers and leaves of *Silybum marianum* (milk thistle), has been widely used as a natural remedy to treat hepatic disorders throughout the world (Vargas-Mendoza et al., 2014). The pharmacological effects of silymarin, specifically in treating chronic and acute liver diseases, drug-induced hepatitis, liver cirrhosis, and even cancer have been demonstrated in a number of experimental and clinical studies (Gazak et al., 2007; Cheung et al., 2010; Neha et al., 2016). Silybin, a mixture of silybin A (SBA) and silybin B (SBB) in a 1:1 ratio, is the most biologically active component of silymarin, which exhibits antioxidant, antifibrotic, anti-inflammatory, membrane stabilizing, and liver regenerating activities (Kvasnicka et al., 2003; Davis-Searles et al., 2005; Loguercio and Festi, 2011). Despite the promising therapeutic potential, the application of silybin is limited by poor absorption, rapid metabolism, and ultimately poor oral bioavailability (Javed et al., 2011). Although novel pharmaceutical formulations such as liposomes and phytosomes could improve the poor water solubility and drug permeability of silybin (Theodosiou et al., 2014), the active concentrations in the plasma of patients who were given a high dosage of Legalon^®^ SIL (Madaus) at 700 mg/day were comparatively lower than the concentrations (between 20 and 50 μg/mL), successfully exhibiting anti-inflammatory, anti-oxidant, and anti-viral activities in experimental studies (Fried et al., 2012), which might contribute to the effectiveness and inconsistent therapeutic results observed in clinical studies (Jacobs et al., 2002; Verma and Thuluvath, 2007). After oral administration, limited human pharmacokinetic (PK) studies showed that silybin is quickly metabolized to their conjugates, primarily forming glucuronides (Wen et al., 2008) and mainly excreted through the biliary tract (Miranda et al., 2008). However, our previous studies found that the gene polymorphism associated with functional enzymatic deficiency of UDP-glucuronosyltransferase 1A1 (UGT1A1), which is the major metabolic enzyme of silybin, has no effects on the PKs of silymarin in patients with liver diseases (Xie et al., 2017). Therefore, we hypothesized that efflux transporters may be one of the key factors influencing the silymarin system exposure, especially on absorption and elimination.

Efflux transporters of the ATP binding cassette (ABC)-containing family, play a key role in the pharmacological behavior of most of the drugs (Schinkel and Jonker, 2003; Marquez and Van Bambeke, 2011). Substrates or inhibitors of these transporters can limit the transport of other molecules, which potentially affect the bioavailability, distribution, and/or elimination by competing for transport (Marquez and Van Bambeke, 2011; Konig et al., 2013; Gupta et al., 2015). We have demonstrated that silybin is a substrate of the efflux transporters breast cancer resistance protein (BCRP, gene symbol ABCG2) and multi-drug resistance protein 2 (MRP2, gene symbol ABCC2) (Yuan et al., 2017). In this study, we wanted to enhance the bioavailability and pharmacodynamic effects of silybin via the inhibition of the efflux transporters BCRP and MRP2 by using baicalein, which was selected based on the primary screening results in our laboratory.

Baicalein (5,6,7-trihydroxyflavone), one of the primary active components of *Scutellari*a species, is a well-known compound that possesses multiple pharmacological functions such as anticancer, antioxidant, anti-inflammatory, cardio-protective, and neuro-protective properties (Woo et al., 2005; Liu et al., 2010; Bie et al., 2017). Baicalein is well absorbed by passive diffusion (40% absolute absorption) and extensively glucuronidated to baicalin and baicalein 6-O-glucuronide by UGT in either the small intestine or the liver *in vivo* (Lai et al., 2003). Notably, baicalein is a p-glycoprotein (P-gp) inhibitor (Kitagawa et al., 2005), increasing the area under the curve (AUC) of nimodipine (a P-gp substrate) in rats because of the inhibition of P-gp efflux and/or CYP3A4-mediated metabolism (Cho et al., 2011). Moreover, baicalin, the glucuronide of baicalein, has also been reported for its interaction with ABC transporters, including BCRP, MDR1, MRP2, MRP3, and MRP4 (Zhang et al., 2007; Kalapos-Kovacs et al., 2015). However, limited studies have been carried out exploring baicalein as an enhancer of bioavailability and therapeutic effects based on its action on efflux transporters.

In this study, we wanted to get an insight into the key role of efflux transporters in affecting the absorption of silybin based on *in vitro* cell model and PK and pharmacodynamic studies by coadministration with baicalein. This might be of particular interest in explaining the activation of baicalein as a potential efflux transporter inhibitor and in providing a therapeutic method for the clinical use of drugs with low-bioavailability.

## Materials and Methods

### Reagents

Silybin, β-glucuronidase (type B-10 from bovine liver), sulfatase (type H-1 from Helix pomatia), Ko143, MK-571, quinidine, cyclosporin A, 5(6)-Carboxy-2′,7′-dichlorofluorescein diacetate (CDCFDA), Rhodamine 123 (Rho123), and naringenin (NG) were purchased from Sigma-Aldrich (St. Louis, MO, United States). Baicalein and baicalin were purchased from MelonePharma (Dalian Melone Biology Technology Co., Ltd., Dalian, China). Polyethylene glycol 400 (PEG400) was purchased from TCI (Tokyo Chemical Industry Co., Ltd., Tokyo, Japan). Cremophor^®^ EL was purchased from Aladdin (Aladdin Industrial Corporation, Shanghai, China). Alanine aminotransferase (ALT), aspartate aminotransferase (AST), alkaline phosphatase (AKP), and superoxide dismutase (SOD) assay kits were purchased from the Nanjing Jiancheng Bioengineering Institute (Nanjing Jiancheng Bioengineering Institute, Nanjing, China). Tumor necrosis factor-α (TNF-α), interleukin-6 (IL-6), and IL-1β ELISA assay kits were purchased from eBioscience (Thermo Fisher Scientific Inc., Vienna, Austria). All other chemicals and reagents were of analytical grade or higher and were commercially available. Serial dilution of the stock solutions with DMSO was prepared to produce working solutions of all compounds. The portion of DMSO in the final reaction solution was less than 1%.

### Bidirectional Transport Assays With Caco-2 and Transfected MDCK II Cells

Human colon epithelial cell line (Caco-2) was purchased from American Type Culture Collection (Manassas, VA, United States) and frozen at -80°C. Wild type MDCK II, MDR1-MDCK II, BCRP-MDCK II, and MRP2-MDCK II cells were kindly supplied by the Netherlands Cancer Institute (Amsterdam, Netherlands). Transport study was carried out with Caco-2 cells, and MDCK II cells were transfected using a previously established method with slight modifications (Yuan et al., 2017). The Caco-2 cells were cultured at 37°C/5% CO_2_ in Dulbecco’s modified Eagle’s medium (DMEM) supplemented with 10% fetal bovine serum, 1% nonessential amino acids, and 1% penicillin-streptomycin. The Caco-2 cells were seeded onto polyester filter membranes in a 12-well Transwell^®^ plate (Corning Costar Co., NY, United States) with 0.6 × 10^5^ cells/cm^2^. The monolayer cells were used for transport assays between 21 and 23 days. The MDCKII cells were seeded on transwell plates with a density of 2.0 × 10^5^ cells/cm^2^ and grown for 4–5 days to form a confluent monolayer.

Bidirectional transport assays for Caco-2 and MDCK II cells were performed by adding silybin (10 μM) in assay buffer into either the apical (AP; A-to-B) or basolateral (BL; B-to-A) chamber, and each 100 μl sample was collected at 30, 60, 90, 120, and 180 min. For inhibition studies, Caco-2 or MDCK II cell monolayers in both apical and basolateral chambers were preincubated with the inhibitor for 30 min. The fluorescence intensity of the sample collected from the AP or BL side was immediately detected in a 96-well black plate by the Microplate UV/VIS spectrophotometer (TECAN Infinite M200 PRO, Männedorf, Switzerland). For liquid chromatography tandem-mass spectrometry (LC-MS/MS) analysis, the sample (100 μl) was mixed with methanol (50 μl) containing internal standard (25 ng/ml) and was stored at -80°C. The formula for calculating apparent permeability coefficient (Papp) values is as follows:

(1) $$P_{app}=\frac{\text{d}Q}{\text{d}t}\times \frac{1}{{\text{C}}_{0}A}$$

where dQ/dt (mM/s) represents the rate of permeation of the drug across the cells, C_0_ (mM) refers to the concentration in the donor compartment at time zero calculated from dosing solution, and A (cm^2^) refers to the area of the cell monolayer. Papp (B-A) is the Papp value measured in the B to A direction, and Papp (A-B) is the Papp value measured in the A to B direction. The efflux ratio is given as ER = Papp (B to A)/Papp (A to B).

### Evaluation of Biliary Excretion Using Rat Sandwich-Cultured Hepatocytes

Sandwich-cultured rat hepatocytes (SCHs) model was established based on a previously described method with minor modifications (Swift et al., 2010). In brief, primary rat hepatocytes were prepared from male Sprague Dawley (SD) rats (220–270 g) using collagenase perfusion as described previously (Liu et al., 1999) and then were plated on 6-well plates at a density of 7.4 × 10^5^ cells/2 ml/well. Approximately 24 h later, hepatocytes were overlaid with 2 ml of BD Matrigel basement membrane matrix in DMEM (0.25 mg/ml) containing 1% (v/v) insulin/transferrin/selenium culture supplement, 0.1 M dexamethasone, 2 mM L-glutamine, and 1% penicillin-streptomycin. Hepatocytes were cultured for 4 days with daily medium replacements until the canalicular network was extensively formatted between the cells before the experiment.

Evaluation of biliary excretion in SCHs was carried out according to a previously described method (Liu et al., 1999). In brief, SCHs were preincubated in warm HBSS (2 ml) with or without Ca^2+^ at 37°C for 15 min. After HBSS (without Ca^2+^) exposure, the tight junctions of the bile canaliculi temporally opened. Sandwich-cultured hepatocytes were further incubated at 37°C for 20 min in 1.5 ml HBSS containing silybin (10 μM) in the absence (control) or presence of the inhibitor (50 μM MK571 as a positive control or baicalein). Subsequently, cells were rinsed with ice-cold HBSS three times and lysed in 1 ml water by sonication for 5 s using the ultrasonic cell disruption system (Sonics & Materials, Inc., Newtown, CT, United States). Samples were quantified by LC-MS/MS analysis, and the transport function was normalized to the protein content of the hepatocytes. The calculation of biliary excretion index (BEI; %) and *in vitro* biliary clearance (Cl_biliary_; ml/min/kg) was based on the following equations powered by B-CLEAR technology (Qualyst, Inc., Research Triangle Park, NC, United States):

CL_bile, *int*_ =

(2) $$\frac{{\text{Uptake(+Ca}}^{\text{2+}}/{\text{Mg}}^{\text{2+}})-\text{Uptake }(-{\text{Ca}}^{2+}/{\text{Mg}}^{\text{2+}})}{\text{Incubation time}\times \text{Concentration (medium)}}$$

BEI% =

(3) $$\frac{{\text{Uptake(+Ca}}^{\text{2+}}/{\text{Mg}}^{\text{2+}})-\text{Uptake }(-{\text{Ca}}^{2+}/{\text{Mg}}^{\text{2+}})}{{\text{Uptake(+Ca}}^{\text{2+}}/{\text{Mg}}^{\text{2+}})}\times 100\%$$

### Quantification of Conjugated Silybin and Free Silybin

A previously established liquid chromatography-mass spectrometry (LC-MS) method with slight modifications was used to determine the concentrations of all glucuronide and sulfate conjugates of silybin and free silybin in bio-samples (plasma, cell and media) (Wen et al., 2008). In brief, a 10 μl aliquot of each sample was enzymatically hydrolyzed with bovine liver (8000 U/ml) and sulfatase (80 U/ml), and the reaction was terminated by adding 40 μl ice-cold acetonitrile containing glacial acetic acid (1%) and internal standard NG (25 ng/ml). After centrifugation at 13,500 rpm for 15 min at 4°C, the supernatant was dried in ice bath by blowing nitrogen. Later, the residues were reconstituted with 60 μl of 30% methanol in water containing 1% ascorbic acid, and an aliquot of 10 μl of final solution after centrifugation was analyzed by LC-MS/MS. In parallel, a 10 μl aliquot of each sample without enzymes was treated with 40 μl ice-cold acetonitrile containing glacial acetic acid (1%) and NG (25 ng/ml) to determine the concentration of free silybin. Therefore, the concentration of total glucuronides and sulfates was estimated based on the difference between the amount of total silybin (after incubation with both glucuronidase and sulfatase) and free silybin (without enzymatic incubation). The method was validated in terms of specificity, recovery, linearity, sensitivity (LLOQ), matrix effect, accuracy, precision, and stability based on the USA Food and Drug Administration (FDA) bioanalytical method validation guidance. The quantitative linear range for silybin A and B was 5 to 12500 ng/ml.

### LC-MS/MS Conditions

The analysis for the determination of the concentration of silybin and its glucuronides and sulfates was performed on an Agilent HP 1100 LC-MS system (Agilent, CA, United States) with Waters ACQUITY UPLC^®^ BEH C18 Column (1.7 μm, 2.1 × 50 mm, Waters Corp., Ireland) protected by a C18 Security Guard cartridge (4 × 2.0 mm i.d., Phenomenex, Torrance, CA, United States). Silybin A and B were well separated using optimized gradient elution with 0.1% formic acid in water (mobile phase A) and methanol (mobile phase B) at a flow rate of 0.4 ml/min within 10 min at room temperature (22°C). The gradient elution was performed as follows (time: mobile phase B percentage): 0 min: 25%, 6 min: 40%, 6.5 min: 25%.

Mass spectrometry parameters: capillary voltage, -4000 V; drying gas, 9 L/min; drying gas temperature, 325°C; nebulizer pressure, 40 psi; fragment voltage, 35 V; dwell time, 200 ms; scan mode, selective ion monitoring (SIM) with [M-H]^-^ for silybin (*m/z* 481), mono-glucuronide (*m/z* 657), and NG (*m/z* 271). The LC-MS data were collected by Agilent ChemStation Software.

### Pharmacokinetic Studies

Male SD rats, weighting 200–250 g, were purchased from the Guangdong Medical Laboratory Animal Center. They were housed as six rats per cage and in a temperature- (21°C) and humidity-controlled (70%) room with a 12 h light/dark cycle with standard diet and water. All animals were allowed to acclimate to the housing conditions for 7 days prior to experimentation. Experimental procedures were approved by the Committee of Guangdong Provincial Hospital of Traditional Chinese Medicine (No # 2017044).

The SD rats were randomly divided into three groups with six rats per group and were given oral administration as follows: (1) silybin at a dosage of 50 mg/kg bodyweight, (2) silybin and baicalein at a dosage of 50 mg/kg each, and (3) baicalein at a dosage of 50 mg/kg body weight. Before the experiment, rats were pre-fasted for 24 h with water *ad libitum* and were maintained at a temperature of 21°C with 70% relative humidity and a 12 h light/dark cycle. Blood samples were collected via the tail vein (about 0.1 ml) at time intervals of 0, 15, 30, 45, 60, 90, 120, 240, 360, 480, and 720 min after oral administration with a temporary cannula (Parasuraman et al., 2010), and the samples were transferred into heparinized micro-centrifuge tubes. Immediately, the blood samples were centrifuged at 4,000 rpm for 15 min at 4°C, and the resulting plasma was isolated and stored at -80°C until analysis.

Pharmacokinetic parameters including half-life (T_1/2_), volume of distribution (Vd), oral clearance (CL/F), and AUC were calculated using the PK Solutions 2.0.2 software package (SUMMIT Co., Ashland, OH, United States). The non-compartment model was adopted for the PK analysis.

### Acute Liver Damage Induced by CCl_4_ in Rats

Male SD rats were randomly divided into seven groups: (1) Group I, normal control given blank vehicle (35% PEG400: 15% Cremophor EL: 5% ethanol : 45% saline) per *os* (p.o.) for 7 days and intraperitoneally (i.p.) injected with olive oil (2 ml/kg body weight) on day 7; (2) Group II, given blank vehicle p.o. for 7 days; (3) Group III, treatment with silybin (100 mg/kg bodyweight, p.o.) for 7 days; (4) Group IV, treatment with silybin (100 mg/kg bodyweight, p.o.) and baicalein (100 mg/kg bodyweight, p.o.) mixture for 7 days; (5) Group V, treatment with silybin (100 mg/kg bodyweight, p.o.) and baicalein (50 mg/kg bodyweight, p.o.) mixture for 7 days; (6) Group VI, treatment with silybin (100 mg/kg bodyweight, p.o.) and baicalein (25 mg/kg bodyweight, p.o.) mixture for 7 days; and (7) Group VII, treatment with baicalein (100 mg/kg bodyweight, p.o.) for 7 days. Rats in Group II-VII were injected with CCl_4_–olive oil mixture (40% CCl_4_, i.p., 2 ml/kg body weight) at 2 h after administration of silybin or/and baicalein on day 7.

After 24 h of CCl_4_ treatment, all rats were sacrificed under ether anesthesia. Blood samples were collected from the abdominal aorta for biochemical estimations. The liver was immediately removed and weighed with a large portion of the liver frozen in liquid nitrogen. The pathological liver changes were examined by hematoxylin and eosin (H&E) staining, which was performed by a pathologist who was blinded to this study. The serum levels of ALT, AST, AKP, and SOD as well as the cytokines TNF- α, IL-6, and IL-1β were detected by the assay kit, according to the manufacturer’s instructions.

### Statistical Analysis

One-way ANOVA was used for the comparison analysis, and the Bonferroni *post hoc* correction was used to accommodate multiple testing. The two-sided unpaired Student’s *t*-test was applied for comparing the differences between the two groups. Data that failed to fall into normal distribution were normalized through log transformation for statistical comparison. During statistical analysis, differences in group sizes were considered in the calculations. Differences were considered statistically significant when *P* < 0.05. All calculated data are presented as mean ± SD.

## Results

### Baicalein Inhibited Efflux Transport of Silybin in Caco-2 Cells

**FIGURE 1:**
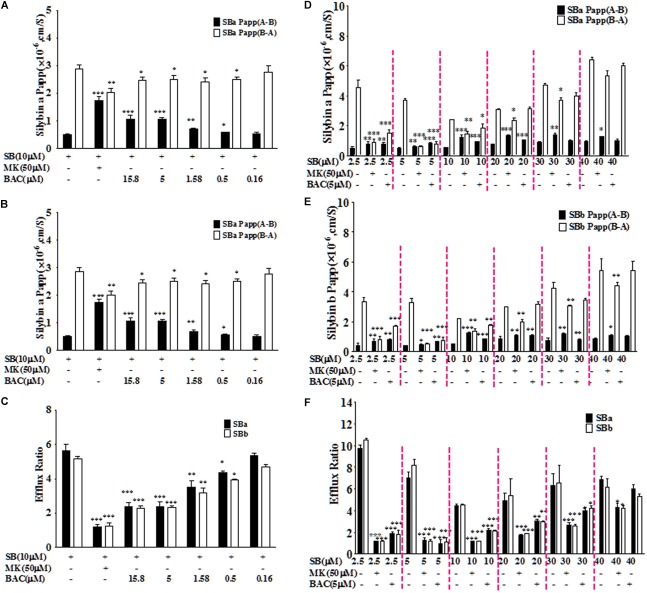
Baicalein inhibited the efflux of silybin across Caco-2 cell monolayers. The effects of baicalein at different concentrations on the permeability values of silybin A (SBA) **(A)** and silybin B (SBB) **(B)** as well as efflux ratio **(C)** of silybin in the Caco-2 cell model. Moreover, inhibition effects of baicalein (5 μM) on the permeability values of SBA **(D)** and SBB **(E)** and efflux ratio **(F)** decreased with the increased concentration of silybin across Caco-2 cell monolayers (^∗^*P* < 0.05; ^∗∗^*P* < 0.01; ^∗∗∗^*P* < 0.001 vs. silybin only, *n* = 3).

Baicalein was selected based on primary screening experiments in Caco-2 cells. The mean efflux ratios of silybin A and B in Caco-2 model were 5.62 and 5.13, respectively, indicating active transport of efflux, which is consistent with the literature (Yuan et al., 2017). As shown in Figures 1A–C, baicalein could inhibit efflux transport of silybin (10 μM) in the Caco-2 cell monolayers with the lowest effective dosage at 0.5 μM. Co-treatment with baicalein (5 μM) could reduce the efflux ratios of silybin A and B to 2.38 ± 0.27 and 2.33 ± 0.09, respectively. We noted that the highest dosage of baicalein (15.8 μM) used in this study revealed a similar effect as that of the dosage at 5 μM, indicating that efflux inhibition of baicalein could be saturated.

Considering the saturation effect observed with different dosages of baicalein, we also evaluated the relationship between the inhibition effects of baicalein (5 μM) and the concentrations of silybin (2.5, 5, 10, 20, 30, and 40 μM). As shown in Figures 1D–F, baicalein at 5 μM could significantly inhibit the efflux of silybin with concentrations below 40 μM. However, the inhibition effects of baicalein decreased with the rising concentration of silybin. Moreover, baicalein could reduce the efflux ratios of silybin (2.5, 5, and 10 μM) to about 2.0, which is similar to the effect observed with MK571 at 50 μM (Figure 1). Therefore, baicalein could significantly inhibit the efflux of silybin in Caco-2 cells.

### Baicalein Inhibited Biliary Excretion of Silybin Conjugates in Rat SCH

**FIGURE 2:**
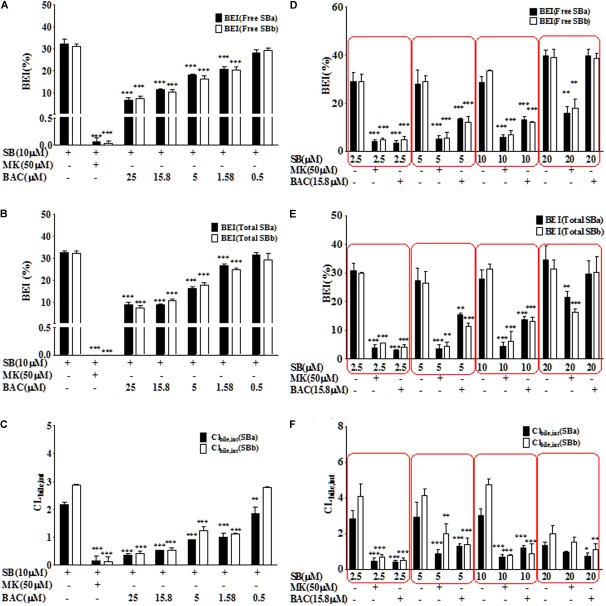
Baicalein inhibited the biliary excretion of silybin and its conjugates in the SCH model. Baicalein inhibited the BEI value of free silybin **(A)** and total silybin **(B)** as well as Cl_bilary,int_ value of free silybin **(C)** in a dose-dependent manner. Moreover, the BEI values of free silybin **(D)** and total silybin **(E)** as well as Cl_bilary,int_ **(F)** reflect the effects of baicalein at 15.8 μM on the excretion of silybin at different concentrations (^∗^*P* < 0.05; ^∗∗^*P* < 0.01; ^∗∗∗^*P* < 0.001 vs. silybin only group, *n* = 3).

After oral administration, extensively glucuronide (55%) and sulfate (28%) conjugated silybin are mainly excreted through the biliary tract (Wen et al., 2008). Considering the major role of MRP2 in the biliary excretion of conjugates, the SCH model was used to study the hepatobiliary transport of silybin conjugates as it has polarized expression of native transporters and liver metabolism enzymes (Swift et al., 2010). The effects of baicalein on the biliary efflux of silybin conjugates were measured and presented as BEI and Cl_biliary_ (Figure 2) by using the B-CLEAR technology in the primary rat SCH model. Accumulation of silybin conjugates in bile was measured either in the absence or presence of Ca^2+^/Mg^2+^ in the extracellular medium, and BEI and Cl_biliary_ were calculated by taking into account the accumulation differences under these conditions. In the absence of baicalein, BEIs for total silybin A or B were 32.7 ± 0.8% and 32.3 ± 1.1%, respectively, after incubation for 20 min with silybin (10 μM) in the rat SCH model, indicating that the biliary transport might play a critical role in the excretion of silybin.

Baicalein had a significant inhibitory effect on BEI of silybin and its conjugates in a dose-dependent manner (Figures 2A,B). Inhibitor of multi-drug resistance proteins (MRPs), MK571, was used as a positive control in this study, because MRP2 was identified as the key transporter mediating the biliary elimination of silymarin flavonolignan conjugates (Miranda et al., 2008). As shown in Figure 2, MK571 (50 μM) significantly inhibited the biliary excretion of silybin and its conjugates. Moreover, baicalein (25 μM) could reduce about ∼90% of the biliary excretion of silybin conjugates (*P* < 0.001). The values of Cl_biliary_ of total silybin in the absence of inhibitor were 2.2 ± 0.1 μl/(min⋅mg protein) for silybin A and 2.9 ± 0.4 μl/(min⋅mg protein) for silybin B. Baicalein significantly decreased the intrinsic Cl_biliary_ values of total silybin compared with the values of silybin alone (*P* < 0.001) (Figure 2C).

As shown in Figure 2D–F, we also studied the relationship between the inhibition effects of baicalein (15.8 μM) and the concentrations of silybin (2.5, 5, 10, and 20 μM). Our results showed that baicalein at 15.8 μM could significantly inhibit the biliary excretion of silybin conjugates (below 10 μM). Moreover, the inhibition effects of baicalein decreased with the rising concentration of silybin.

### Inhibition of Efflux Transporter(s) by Baicalein in Transfected MDCKII Cells

**FIGURE 3:**
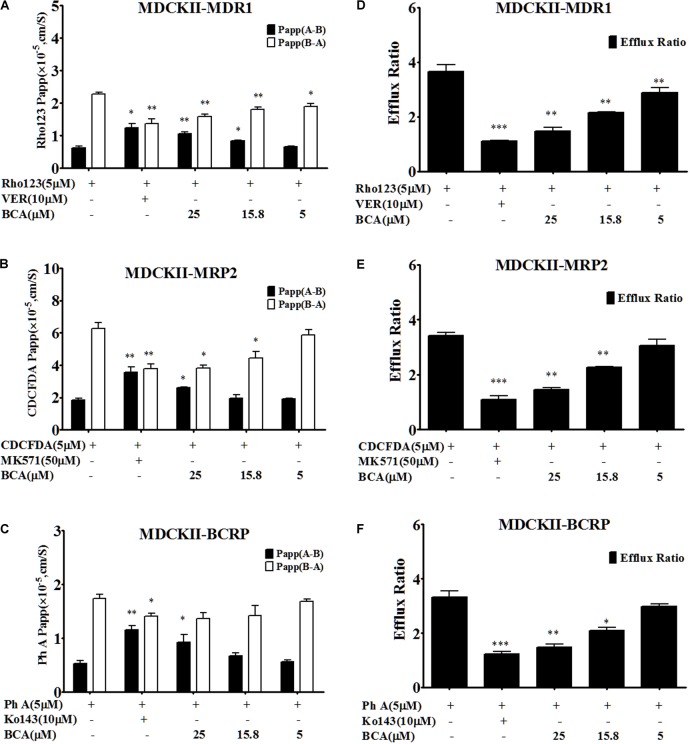
Baicalein is an inhibitor of MDR1, MRP2, and BCRP in MDCKII-transfected (MDR1, MRP2, and BCRP) cell lines. The permeability values **(A)** and corresponding efflux ratios **(D)** of Rho123 (a specific substrate of P-gp) obtained in MDCK II-MDR1 in the absence and presence of baicalein; the permeability values **(B)** and corresponding efflux ratios **(E)** of CDCFDA (a specific substrate of MRP2) obtained in MDCKII-MRP2 in the absence and presence of baicalein; the permeability values **(C)** and corresponding efflux ratios **(F)** of Ph A (a specific substrate of BCRP) obtained in MDCK II-BCRP in the absence and presence of baicalein (^∗^*P* < 0.05;^∗∗^*P* < 0.01; ^∗∗∗^ *P* < 0.001 vs. absence of inhibitor, *n* = 3).

Understanding the interactions between efflux transporter(s) and baicalein could help to illustrate the drug-drug interaction mechanism as silybin is a substrate of both MRP2 and BCRP (Yuan et al., 2017). Transport experiments were conducted across transfected MDCK-II (MDCKII-MDR1, MDCKII-MRP2, and MDCKII-BCRP) cell lines, which were recommended as *in vitro* models for the determination of the extent of transporter inhibition (Agarwal et al., 2007; Giacomini et al., 2010). The efflux ratio of the specific MRP2 substrate CDCFDA was 3.82 ± 0.51 in the MDCKII-MRP2 cells, and it decreased to 1.11 ± 0.19 in the presence of MK571. As shown in Figure 3, the efflux ratio of CDFDA was decreased to 1.46 ± 0.08 by the treatment of baicalein at 25 μM in the MDCKII-MRP2 cells, suggesting that baicalein was an MRP2 inhibitor.

The efflux ratio for pheophorbide A (Ph A, a BCRP-specific substrate) was 2.82 ± 0.13 in the MDCKII-BCRP cells, and it decreased to 0.99 ± 0.09 in the presence of Ko143 (Figure 3). However, the treatment of baicalein inhibited the efflux of Ph A and decreased the efflux ratio to 1.48 ± 0.14, indicating that baicalein was also a BCRP inhibitor.

The MDCKII-MDR1 cell monolayer model was validated by examining the bidirectional transport of Rho123. A predominantly B-A transport of Rho123 was observed with mean efflux ratio at 3.68 ± 0.44, which was significantly reduced by 10 μM verapamil (MDR1 inhibitor), demonstrating the suitability of the MDCKII-MDR1 cell monolayer for investigating the function of P-gp *in vitro*. The efflux ratio for Rho123 in the MDCKII- MDR1 cells was decreased to 1.49 ± 0.21 by adding baicalein (Figure 3), indicating that baicalein was also an MDR1 inhibitor, which was consistent with the literature (Kitagawa et al., 2005).

**FIGURE 4:**
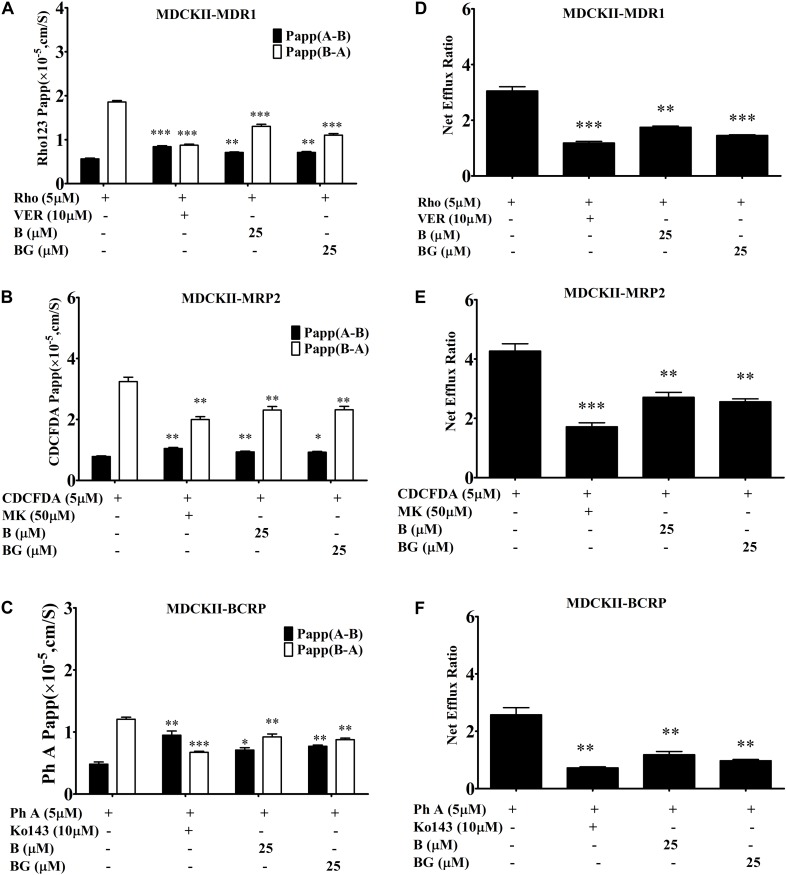
Comparing the inhibition effects of baicalin (BG) and baicalein (B) on efflux transporters across MDCKII-transfected (MDR1, MRP2, and BCRP) cell lines. The permeability values **(A)** and corresponding efflux ratios **(D)** of Rho123 obtained in MDCK II-MDR1 in the absence and presence of baicalein or baicalin; the permeability values **(B)** and corresponding efflux ratios **(E)** of CDCFDA obtained in MDCKII-MRP2 in the absence and presence of baicalein or baicalin; the permeability values **(C)** and corresponding efflux ratios **(F)** of Ph A obtained in MDCK II-BCRP in the absence and presence of baicalein or baicalin (^∗^*P* < 0.05;^∗∗^*P* < 0.01; ^∗∗∗^*P* < 0.001 vs. absence of inhibitor, *n* = 3).

In addition, it has been reported that baicalin, the glucuronide of baicalein, could inhibit efflux transporters, including BCRP and MRP2 (Kalapos-Kovacs et al., 2015). Here, we also compared the inhibition effects of baicalein and baicalin in transfected MDCK-II cell lines. As shown in Figure 4, both baicalein and baicalin had similar inhibition effects on MDR1, MRP2, and BCRP. Therefore, both baicalein and its *in vivo* metabolite baicalin could inhibit the efflux transport of silybin, which might improve *in vivo* bioavailability and boost hepatic-protective effects.

### Coadministration of Baicalein Enhances the Absorption of Silybin in Rats

Next, we wanted to investigate the effect of baicalein on the PKs of silybin *in vivo*. A validated analytical method for the determination of silybin and its total conjugates in rat plasma by LC-MS was developed based on a previous report with minor modifications (Yuan et al., 2017). No endogenous interfering peak was observed in the plasma at retention times of the analytes and internal standard NG by using the highly selective SIM detection mode. The calibration curves showed a correlation coefficient (r^2^) of 0.999 for silybin A & B in plasma as presented in Supplementary Table S1. The results of precision and accuracy for intra- and inter-day evaluation are presented in Supplementary Table S2. The lower limit of quantification (LLOQ) was 5 ng/ml with relative standard deviation (RSD) ≤6.4%. The RSD values of quality control (QC) samples examined for precision were within 6.4%, while the accuracy ranged from 91.7 to 109.5%. It can be seen from Supplementary Table S2 that the extraction recoveries of silybin A and B were above 84.4% in plasma. Stability of SBA and SBB in plasma was also tested using QC samples at four different concentrations in the stability tests of three freeze–thaws, long-term, short-term, and post-preparative. No impairment of quantification was observed for the samples of stability testing. These results (Supplementary Table S3) suggested that samples could be handled under normal laboratory conditions without significant loss of SBA and SBB.

**FIGURE 5:**
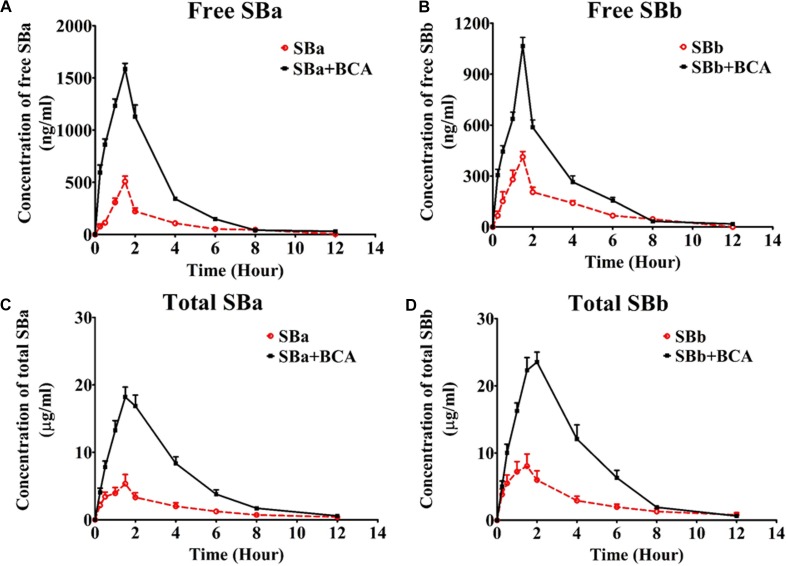
Mean plasma concentration-time profiles of silybin in rats after a single oral administration of silybin (50 mg/kg) or in combination with baicalein (50 mg/kg) (*n* = 6). **(A)** free silybin A, **(B)** free silybin B, **(C)** total silybin A and **(D)** total silybin B average plasma concentration time curves were illustrated. Total SBA or total SBB means total amount of free silybin and their conjugated glucuronides and sulfates.

The mean plasma concentration-time profiles of silybin following an oral administration (50 mg/kg) in the presence or absence of baicalein (50 mg/kg) are shown in Figure 5, and the calculated PK parameters of silybin are summarized in Table 1. In comparison with silybin given alone (50 mg/kg), coadministration with baicalein (50 mg/kg) effectively enhanced the plasma concentration of free silybin (Figure 5) and increased the maximum concentrations (C_max_) of silybin A and B increased from 508 ± 114 to 1526 ± 126 and 413 ± 69 ng/ml to 915 ± 150 ng/ml, respectively Homogeneously, AUC_(0-t)_ of free silybin A and B were increased from 1292 ± 341 to 3534 ± 344 and 1195 ± 302 to 1808 ± 225 ng^∗^h/ml, respectively. No significant difference in T_max_ was found among the groups studied.

**Table 1:** Mean pharmacokinetic parameters of silybin at a dose of 50 mg/kg in the absence and presence of 50 mg/kg baicalein in rats.

	Parameters^A^	Without Baicalein		With Baicalein	
		SBA	SBB	SBA	SBB
Free silybin	AUC_(0-t)_ (ng ⋅ h/ml)	1292 ± 342	1195 ± 302	3534 ± 344^***^	1808 ± 226^**^
	AUC_(0-∞)_ (ng ⋅ h/ml)	2305 ± 1147	2087 ± 932	3599 ± 410^*^	1812 ± 223
	MRT_(0-t)_ (h)	4.1 ± 0.9	4.21 ± 0.98	1.9 ± 0.2	2.1 ± 0.3
	t_1/2z_(h)	1.5 ± 0.2	1.7 ± 0.3	1.6 ± 2.3	1.34 ± 0.53
	T_max_(h)	1.50 ± 0	1.50 ± 0	1.5 ± 0	1.29 ± 0.51
	CL_z/F_(l/h/kg)	25.0 ± 8.5	27.0 ± 8.6	14.0 ± 1.6	17.9 ± 3.7
	C_max_(ng/ml)	507.7 ± 115	412.7 ± 69.3	1526 ± 126^***^	915.2 ± 151^***^
Total silybin	AUC_(0-t)_ (ng ⋅ h/ml)	12450 ± 2553	53184 ± 7340	25921 ± 2277^***^	97584 ± 23856^**^
	AUC_(0-∞)_ (ng ⋅ h/ml)	14475 ± 4645	54680 ± 7924	26906 ± 3129^***^	98637 ± 24179^**^
	MRT_(0-t)_ (h)	3.7 ± 0.4	3.7 ± 0.3	3.8 ± 0.2	3.2 ± 0.2
	t_1/2z_(h)	2.1 ± 0.9	1.9 ± 0.9	3.1 ± 2.6	2.6 ± 0.2
	T_max_(h)	1.3 ± 0.3	1.6 ± 0.4	1.8 ± 0.3	1.8 ± 0.3
	CL_z/F_(l/h/kg)	1.9 ± 0.2	0.9 ± 0.1	0.8 ± 0.2	0.6 ± 0.2
	C_max_(ng/ml)	6427 ± 2284	9668 ± 2134	18194 ± 3634^**^	23522 ± 3643^***^

*^a^: C_max_, maximum plasma concentration; T_max_, time to reach maximum plasma concentration; T_1/2_, half-life; MRT, mean residence time; CL/F, oral clearance; AUC_0-t_, area under the concentration-time curve from zero up to a definite time t; AUC_0-∞_, area under the concentration-time curve from zero up to infinite time.*

*^b^Data are expressed as the mean ± SD of n = 6 rats. ^∗^P < 0.05; ^∗∗^P < 0.01; ^∗∗∗^P < 0.01 vs. silybin only.*

Absorption of total silybin, including all glucuronide and sulfate conjugates and free silybin, were also evaluated at the same time. Oral coadministration of silybin with baicalein resulted in a significant increase in C_max_ of total silybin A and B by 283% and 243%, respectively, while AUC_(0-t)_ of total silybin A and B increased from 12450 ± 2553 to 25921 ± 2277 and 53184 ± 7340 to 97584 ± 23856 ng^∗^h/ml, respectively. Considering the wide therapeutic window of silybin (Fried et al., 2012), increased bioavailability of silybin might lead to enhanced therapeutic efficacy without side effects.

### Boosted Hepatoprotective Effects of Silybin in CCl_4_-Induced Acute Liver Injury Model

**FIGURE 6:**
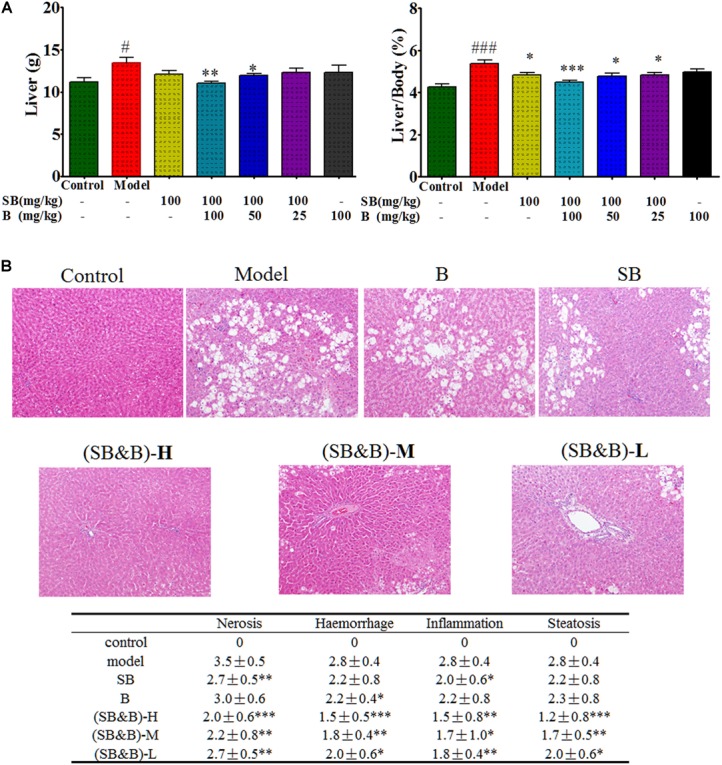
Liver weight and liver to body weight ratio as well as histologic images of liver samples of CCl_4_-induced liver injury model. **(A)** Liver weight and liver to body weight ratio after oral administration of silybin in the absence and presence of baicalein in the liver tissues. **(B)** Histologic images of liver samples. (SB&B)-H, silybin 100 mg/kg and baicalein 100 mg/kg; (SB&B)-M, silybin 100 mg/kg and baicalein 50 mg/kg; (SB&B)-L, silybin 100 mg/kg and baicalein 25 mg/kg. Values are mean ± SD, *n* = 6 in each group. ^∗^*P < 0.05*, ^∗∗^*P* < 0.01, and ^∗∗∗^*P* < 0.001 vs. model group; ^#^*P < 0.05*, ^###^*P* < 0.001 vs. control group.

Carbon tetrachloride (CCl_4_)-induced liver injury is a classic model of chemical liver injury, and it was used in this study to explore the preventive and therapeutic effects of silybin in combination with baicalein. The body weight of rats ranged from 245–260 mg, which was similar among each group. As shown in Figure 6A, the weight of liver and the ratio of liver to body weight were elevated after CCl_4_-treatment compared with those of normal rats, while these increases were abrogated in silybin-treated and silybin and baicalein co-treated groups (*p* < 0.01). Livers of rats in the normal control group had clear lobular architecture with the hepatic cells arranged in neat rows by H&E staining as shown in Figure 6B. However, after CCl_4_ treatment, the livers of the rats in the model group became larger with pale and irregular surface. Moreover, we observed round necrotic lesions around the hepatic lobule portal area with clear nerosis, hemorrhage, inflammation, and steatosis, suggesting severe hepatocellular damage. The liver injury evaluated by histopathological analysis was less prominent in silybin-treated animals. Notably, CCl_4_-induced macroscopic and histopathological changes were significantly attenuated in silybin and baicalein co-treated animals by comparing with that of silybin-treated as shown in Figure 6B.

**FIGURE 7:**
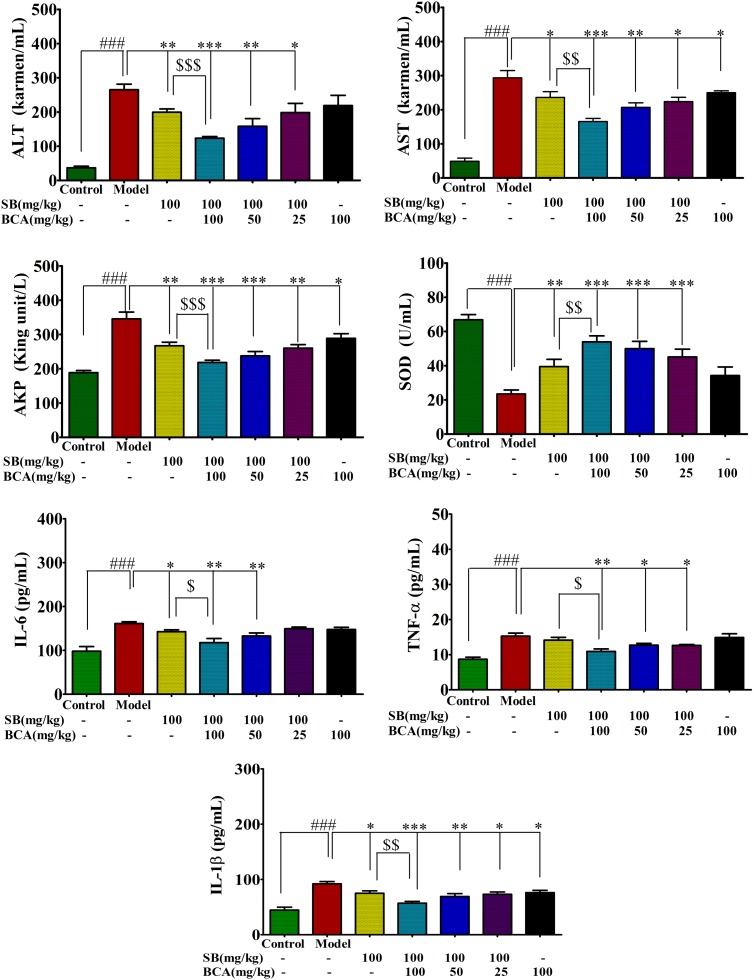
Effect of silybin coadministration with baicalein on CCl_4_-induced changes in serum liver enzyme activities (ALT, AST, and AKP), hepatic oxidative stress SOD, and inflammatory response (IL-1β, IL-6, and TNF-α levels) in rats. Values are mean ± SD, *n* = 6 in each group. ^∗^*P < 0.05*, ^∗∗^*P* < 0.01, and ^∗∗∗^*P* < 0.001 vs. model group; ^###^*P* < 0.001 vs. control group. ^$^*P* < 0.05, ^$$^*P* < 0.01, and ^$$$^*P* < 0.001 vs. SB given alone.

To evaluate the effects of coadministration of silybin and baicalein on liver injury induced by CCl_4_ in rats, the serum levels of ALT, AST, and AKP were measured. As shown in Figure 7, after acute CCl_4_ challenge, the serum levels of ALT and AST in CCl_4_-treated group increased by 5 to 6 times over those in the control group. Moreover, our results showed that the cytokines IL-1β, IL-6, and TNF-α in the CCl_4_-treated group were significantly increased compared with those of the control group as an index of inflammatory response in liver (Figure 7). Pre-administration of silybin at 100 mg/kg for 7 consecutive days significantly prevented the CCl_4_-induced increase in the serum activities of ALT, AST, and AKP (*P < 0.01*) as well as serum IL-1β, IL-6, and TNF-α levels (*P < 0.01*). However, at the same dosage, coadministration of baicalein with silybin showed a boosted hepatoprotective effect than that seen in the case of silybin given alone (Figure 7).

As antioxidant properties of silybin are considered to be responsible for its hepatoprotective actions, we also evaluated the activity of the antioxidant enzyme SOD as an indicator of oxidative stress in rats with CCl_4_-induced liver injury. As shown in Figure 7, CCl_4_ exposure induced a remarkable decrease in hepatic activities of SOD compared with that of the control group. These changes were remarkably ameliorated in silybin-baicalein co-treated group compared with the CCl_4_ group (*P* < 0.001).

Therefore, coadministration of baicalein with silybin boosted liver protective, antioxidant, and anti-inflammatory effects in the CCl4-induced liver injury model compared with silybin alone, which is related with the enhanced plasma concentration of silybin by the inhibition of BCRP and MRP2.

## Discussion

Based on literature reports, silybin seems to be a promising drug for the treatment of chronic liver disease with clearly demonstrated anti-fibrotic, antioxidant, and metabolic effects in experimental studies. The good safety profile (doses <10 g/d have no significant side effects), standardization, and easy availability are added advantages to its promising therapeutic effects (Pradhan and Girish, 2006). However, previous human studies of silybin were insufficient to confirm the clinical efficacy in chronic liver disease because of no definitive results in terms of clinical efficacy, which might be caused by its poor absorption and low bioavailability (Loguercio and Festi, 2011). Therefore, bioavailability is a major hurdle in the translation of the preclinical potential of silybin. Our previous study found that UGT1A1 is the major metabolic enzyme for silymarin, but UGT1A1^∗^28 polymorphism with reduced UGT1A1 activity does not appear to affect silymarin exposures in patients with chronic hepatitis C virus (HCV) infection, indicating that the metabolic enzyme might have limited effects on the PK behavior of silymarin. Therefore, in this study, we wanted to evaluate the role of efflux transporters on the PK of silybin by using a multiple efflux transporter inhibitor baicalein. The present PK study clearly demonstrates that the coadministration of baicalein markedly increased the absorption of silybin that was quantified with AUC and C_max_ in rats. Moreover, the boosted bio-efficacy of silybin was observed by coadministration with baicalein in the CCl_4_-induced acute liver injury model with enhanced anti-inflammatory and antioxidant effects by comparing with silybin given alone. Baicalein itself failed to show significant benefits in the protection of live injury. These findings suggested that baicalein increased the bioavailability of silybin leading to enhanced hepatoprotective effects via the inhibition of the efflux transporters MRP2 and BCRP. However, synergistic effects observed in our study for silybin and baicalein could not only be related with the enhanced absorption but also caused by the direct pharmacological synergistic effects as both baicalein and silybin have anti-inflammatory and antioxidant activities.

Both baicalein and baicalin (a glucuronide of baicalein) are bioactive constituents of *Scutellariae radix* with various beneficial activities such as the modulation of efflux transporters (Srinivas, 2010; Bie et al., 2017; Sowndhararajan et al., 2017). After oral administration or dosing in the Caco-2 cell model, baicalein underwent a fast and extensive phase II metabolism with predominant glucuronides/sulfates of baicalein (75.7%) in the plasma or cell (Lai et al., 2003; Li et al., 2012). The enhanced bioavailability of silybin might be caused by the baicalin generated after oral administration of baicalein as baicalin has been demonstrated to be an inhibitor of MRP2 and BCRP (Kalapos-Kovacs et al., 2015). But the effects of baicalein on the efflux transporters MRP2 and BCRP is unknown. In this study, we have characterized and compared the effects of baicalin and baicalein on efflux transporters in transfected MDCKII cells with MRP2, BCRP, or P-gp overexpression. The results showed that both baicalein and baicalin are the inhibitors of MRP2, BCRP, and P-gp. However, after oral administration, the relative absorption for baicalin was 65% when compared with baicalein (Lai et al., 2003). Therefore, the use of baicalein was suggested as a combination therapy method for silybin instead of baicalin.

In this study, our results demonstrated that baicalein inhibits the efflux of silybin and improves the intestinal absorption. Therefore, both free and conjugated silybin glucuronides increased in plasma, which is consistent with the observations from the *in vitro* experiment. But the enhanced absorption might also be contributed by the metabolism, although previous studies carried out with UGT1A1^∗^28 polymorphism with reduced UGT1A1 activity did not appear to affect silymarin exposures. Baicalein undergoes quick phase II metabolism, which is similar to that observed with silybin after absorption. After coadministration, baicalein might inhibit the conjugation of silybin by competing with UGT or/and sulfotranferase (SULT). We are currently working on this hypothesis. Moreover, baicalein dramatically decreased biliary clearance and BEI of silybin conjugates in primary liver cells. As glucuronide of silybin is deconjugated readily by rat liver β-glucuronidase, inhibition of biliary excretion of silybin conjugates could increase the concentration of conjugates as well as free silybin, leading to increased circulation concentration of silybin in addition to its therapeutic effects.

Both *in vivo* and *in vitro* studies demonstrated that baicalein improves the bioavailability and therapeutic effects of silybin via the inhibition of the efflux transporters, including MRP2 and BCRP. Combination therapy with silybin and baicalein may be a good choice for the clinical treatment of chronic liver diseases.

## Author Contributions

YX, HZ, and LL participated in research design. PX, Z-WY, and Y-ZL conducted the experiments. PX, YX, and Z-WY performed the data analysis. YX and C-XL wrote or contributed to the writing of the manuscript.

## Conflict of Interest Statement

The authors declare that the research was conducted in the absence of any commercial or financial relationships that could be construed as a potential conflict of interest.

## Abbreviations

AUC: area under the plasma concentration-time curve
BCRP: breast cancer resistance protein
BEI: biliary excretion index
CCl_4_: carbon tetrachloride
Cl_biliary_: biliary clearance
HCV: hepatitis C virus
HE: hematoxylin and eosin
LC-MS/MS: liquid chromatography tandem-mass spectrometry
LLOQ: lower limit of quantification
MRPs: multi-drug resistance proteins
SBA: Silybin A
SBB: silybin B
SCH: sandwich-cultured rat hepatocyte
SOD: superoxide dismutase, UGT1A1-competent
WT: wild type phenotype
