# Supplementary material for: Baicalein Enhances the Oral Bioavailability and Hepatoprotective Effects of Silybin Through the Inhibition of Efflux Transporters BCRP and MRP2
Source: Front Pharmacol. 2018 Oct 26;9:1115. doi: 10.3389/fphar.2018.01115 (PMC6212553; doi:10.3389/fphar.2018.01115)
Supplement: Supplementary file 1 [file Table_1.PDF]

S. Table 1. Calibration curves for determination of SBa, and SBb in plasma samples.

Cpd.	Standard curves	R2	Range
SBa	Y = 0.0002X +0.002	0.9992	5-12500 ng/ml
SBb	Y = 0.0002X- 0.0008	0.9990	5-12500 ng/ml

S. Table 2. Precision, accuracy and recovery for determination of SBa, and SBb in plasma samples (n = 6).

	Conc.	Recovery	Intra-day		Inter-day	
	(ng/ml)	(%,x±SD)	Accuracy (%)	Precision (%)	Accuracy (%)	Precision (%)
SBa	5	94.8±7.0	94.2±3.0	4.5±0.1	98.7±6.3	6.4±2.4
	150	84.4±2.3	109.5±5.7	5.3±1.1	110.2±4.0	1.6±0.8
	750	87.4±6.8	106.9±2.6	2.1±1.3	102.6±6.0	2.3±0.9
	3000	105.7±7.1	91.8±3.1	1.7±0.6	100.2±9.3	1.2±0.1
SBb	5	90.0±7.7	102.9±6.9	4.2±2.1	103.2±5.4	3.7±1.4
	150	88.2±8.5	103.2±5.4	3.0±2.7	105.1±4.6	2.1±1.7
	750	91.6±3.4	102.2±3.5	4.3±2.9	104.6±5.4	1.6±1.0
	3000	103.4±6.8	91.7±1.2	1.6±0.6	99.0±11.5	3.3±2.9

S. Table 3. Stability testing results for determination of SBa, and SBb in plasma samples (n = 3).

		Concentration(x±SD)				
		Initial [a]	Freeze-thaw [b]	Short-term [b]	Long-term [b]	Post-Prep. [b]
SBa	5	4.9±0.1	4.9±0.1	5.2±0.2	5.0±0.1	4.8±0.2
	150	155.6±4.0	155.7±5.2	156.5±4.4	154.0±5.2	157.9±11.2
	750	769.4±10.6	756.1±12.3	736.7±7.3	747.8±12.13	757.8±13.3
	3000	3029±63	2807±43	3046±107	2912±175	3090±71
SBb	5	5.2±0.1	5.1±0.3	5.3±0.1	5.0±0.2	5.4±0.2
	150	163.2±11.4	165.0±8.4	154.1±2.0	149.5±5.7	154.2±3.4
	750	761.4±33.1	718.9±7.5	738.6±36.5	742.8±61.9	738.33±6.3
	3000	2958±127	2834±86	3012±92	2942±182	3027±55

[a]. Contents of silybin and tangeretin in the freshly prepared QC samples. [b]. Contents of silybin and tangeretin in the samples for three freeze–thaw cycles, the long-term, the short-term, and the post-preparation stabilities.
